# Association of serum calcium level with periodontitis: a cross-sectional study from NHANES 2009–2014

**DOI:** 10.3389/fnut.2024.1520639

**Published:** 2025-01-07

**Authors:** Hongliang Cao, Min Wang, Mengna Duan, Song Wang, Haiyang Zhang

**Affiliations:** ^1^Department of Urology II, The First Hospital of Jilin University, Changchun, China; ^2^Department of Prosthodontics, Hospital of Stomatology, Jilin University, Changchun, China

**Keywords:** serum calcium, periodontitis, NHANES, cross-sectional study, epidemiology, nutrition

## Abstract

**Purpose:**

This study aimed to investigate the association between serum calcium levels and periodontitis in a U.S. adult population, using data from the National Health and Nutrition Examination Survey (NHANES) 2009–2014.

**Method:**

Data were analyzed from 8,601 participants aged over 30 years, who were categorized based on the presence or absence of periodontitis. Serum calcium levels were measured using standardized NHANES protocols, and periodontitis status was determined through clinical oral examinations. To assess the relationship between calcium levels and periodontitis, multivariate logistic regression models were applied across three levels of adjustment. Additionally, trend tests and subgroup analyses were conducted to explore associations across different demographic and clinical subgroups. A smoothing curve fitting and threshold effect analysis were also performed to examine potential nonlinear relationships.

**Results:**

After adjusting multiple covariates, participants in the highest quartile of serum calcium showed an 18% reduced risk of periodontitis compared to those in the lowest quartile (OR = 0.82, 95% CI: 0.71–0.95, *p* = 0.0083; *p* for trend = 0.0057). The association remained stable across various subgroups. Smoothing curve fitting indicated a nonlinear negative correlation between calcium levels and periodontitis, though without a significant inflection point at 2.48 mmol/L (*p* = 0.094).

**Conclusion:**

Elevated serum calcium levels appear to be associated with a lower risk of periodontitis in adults. These findings suggest that adequate calcium intake may play a role in periodontitis prevention, providing valuable insight for clinical guidance on nutritional and preventive strategies in periodontal health.

## 1 Introduction

Periodontitis, a chronic inflammatory disease of the periodontium, leads to the progressive destruction of the supporting structures of teeth, including the gingiva, periodontal ligament, and alveolar bone (1–3). It is among the most prevalent dental diseases globally, with its occurrence varying significantly across different age groups, regions, and socioeconomic backgrounds (4–6). Clinically, periodontitis presents with symptoms such as gum swelling, bleeding, pocket formation, and, in severe cases, tooth loss. Despite advances in diagnostic techniques and treatment approaches, the current management of periodontitis remains challenging, particularly in terms of early detection, effective long-term treatment, and sustainable prevention (7–9). There are limitations in the existing diagnostic markers and therapeutic options, which emphasize the need for continued research to better understand the pathophysiology of periodontitis and explore novel preventative and therapeutic strategies (10, 11).

Calcium, an essential mineral in the human body, plays a critical role in numerous physiological processes, including bone mineralization and structural integrity, signal transduction in muscle contraction and neurotransmitter release, enzymatic activation as a cofactor in processes like blood clotting and metabolic regulation (12–14). Calcium deficiency can lead to various health issues, such as osteoporosis, muscle cramps, and cardiovascular complications, reflecting its importance in maintaining overall health (15–17). Previous epidemiological evidence suggested that calcium may also play a significant role in periodontal health (18–20). Studies indicate that adequate calcium levels might be protective against periodontitis, although the underlying mechanisms remain unclear (21, 22). A review highlighted the role of calcium and vitamin D supplementation as a non-surgical adjunctive treatment for periodontal disease, with an emphasis on female patients. Vitamin D facilitates calcium-phosphate homeostasis and bone metabolism, essential for regenerative periodontal therapies (23). This potential link between calcium and periodontitis has attracted research interest as it offers a promising avenue for exploring preventative measures against this disease.

The National Health and Nutrition Examination Survey (NHANES) database, a comprehensive resource for epidemiological studies, provides valuable data on various health conditions and nutritional factors among the U.S. population (24). The purpose of this study is to investigate the relationship between calcium intake and periodontitis using NHANES data, with the aim of offering insights that could contribute to the clinical diagnosis, prevention, and treatment of periodontitis. This study seeks to provide a scientific basis for further exploration of calcium’s role in periodontal health and guide future clinical approaches to managing periodontitis effectively.

## 2 Materials and methods

### 2.1 Study population

This study analyzed data from the NHANES database collected between 2009 and 2014. NHANES is a nationally representative, stratified, large-scale cross-sectional survey, conducted in biennial cycles to evaluate the health and nutritional status of both adult and pediatric populations in the United States. The database includes comprehensive modules on demographic characteristics, dietary intake, physical examinations, laboratory measurements, and questionnaires (24). For additional information, please refer to the NHANES website: https://www.cdc.gov/nchs/nhanes/index.htm.

**Figure 1:**
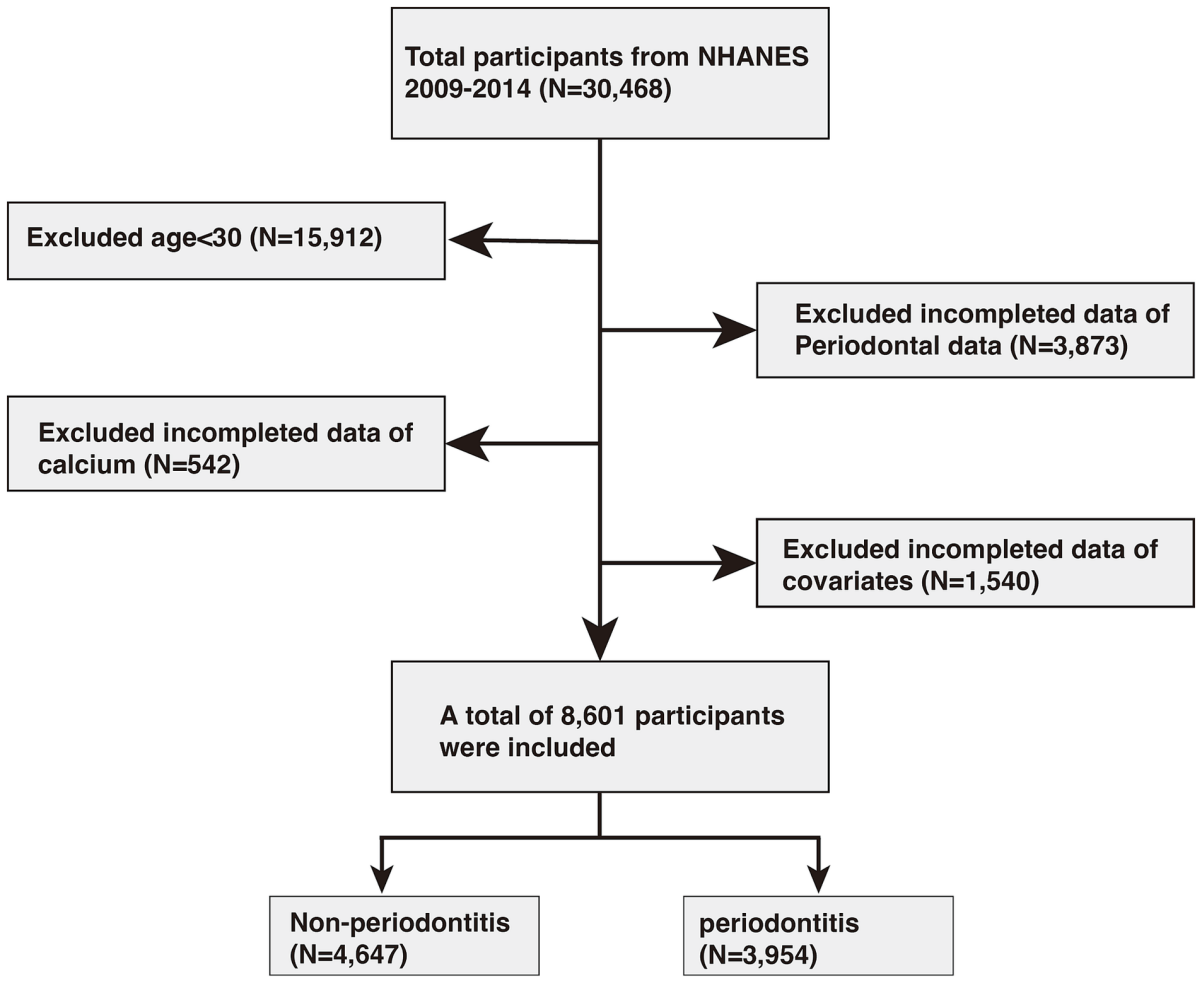
Flowchart of participants selection. NHANES, National Health and Nutrition Examination Survey.

In this study, data were initially collected from 30,468 individuals. Of these, 15,912 participants under the age of 30 were excluded. Additionally, 3,873 individuals were removed due to unavailable periodontal data, while another 542 were excluded due to missing calcium information. A further 1,540 participants were omitted due to incomplete data on other covariates. Ultimately, 8,601 participants met the inclusion criteria, comprising 4,647 individuals without periodontitis and 3,954 with periodontitis. Detailed information can be found in Figure 1.

### 2.2 Definition of exposure

In the NHANES, serum calcium level was measured through biochemical analysis of serum samples collected from participants. Blood samples were typically drawn at the NHANES Mobile Examination Center (MEC) to maintain standardization and ensure high data quality. Calcium measurement is performed using indirect (or diluted) I.S.E. (ion selective electrode) methodology of the DxC800 system to measure calcium concentration in serum. To ensure data accuracy and reliability, NHANES implements rigorous quality control measures, including regular calibration, repeated testing, and verification procedures. For further details on the laboratory methods and quality control processes, the NHANES website provides comprehensive documentation.

### 2.3 Definition of outcome

The primary outcome of this study was the presence of moderate to severe periodontitis. Periodontitis was diagnosed using oral examination data gathered by licensed dentists at the mobile examination center (MEC). To ensure data reliability, dental examiners underwent comprehensive training and calibration, with continuous monitoring and periodic recalibration to maintain data quality standards. The periodontal assessment followed the Full-Mouth Periodontal Examination (FMPE) protocol, measuring attachment loss (AL) and probing pocket depth (PPD) at six specific sites on each tooth, excluding the third molars. AL represents the distance from the cemento-enamel junction to the sulcus base, while probing PPD is the depth from the gingival margin to the sulcus base. Healthy or mild periodontitis were considered as case control group and moderate or severe periodontitis were identify as case group (25, 26). Periodontitis classification adhered to the CDC/AAP criteria, as outlined in Supplementary Table 1 (27).

### 2.4 Consideration of covariates

This study accounted for several potential confounding variables, including age, gender, race, education level, marital status, family poverty-to-income ratio (PIR), smoking status, alcohol, hypertension, diabetes, body mass index (BMI), total protein (TP), total cholesterol (TC), triglycerides (TG), high-density lipoprotein cholesterol (HDL-C), alanine aminotransferase (ALT), aspartate aminotransferase (AST), blood urea nitrogen (BUN), serum uric acid, and serum creatinine (Scr). PIR was categorized into three levels: low (≤1), middle (1–4), and high (≥4). Participants were identified as smokers if they reported having smoked at least 100 cigarettes in their lifetime. Alcohol drinkers were defined as those who consumed at least 12 alcoholic beverages in the past year. Hypertension status was confirmed if participants responded “Yes” to having been told they had high blood pressure. Diabetes status was classified as diabetic, non-diabetic, or borderline based on responses to whether a doctor had ever diagnosed them with diabetes. Detailed information regarding laboratory data collection methods is available on the NHANES website.

### 2.5 Statistical analyses

Statistical analyses in this study adhered to CDC guidelines, incorporating appropriate NHANES sample weights and adjusting for the complex multistage survey design. Participant characteristics, stratified by periodontitis status, were compared using chi-square tests for categorical variables and t-tests for continuous variables. To assess the association between calcium levels and periodontitis, three multivariate logistic regression models were constructed. Model 1 included no covariate adjustments. Model 2 adjusted for age, gender, and race, while Model 3 included a more comprehensive adjustment for factors such as age, sex, race, educational level, marital status, PIR, smoking status, alcohol intake, hypertension, diabetes, BMI, TC, TG, HDL-C, ALT, AST, BUN, SUA, and SCr. Serum calcium levels were further divided into quartiles to apply trend tests, analyzing linear trends in the association between calcium levels and periodontitis. Subgroup analyses were conducted using stratification variables, including age, gender, race, education, marital status, PIR, smoking, alcohol, hypertension, and diabetes, with interaction tests to verify the consistency of associations across these subgroups. Additionally, smoothing curve fitting was applied to explore potential nonlinear relationships between calcium levels and periodontitis. All statistical procedures were performed using R Studio (version 4.3)^1^ and EmpowerStats software (version 2.0)^2^ (see text footnote 1). A *p*-value <0.05 was regarded as statistically significant.

## 3 Results

### 3.1 Baseline characteristics of study participants

A total of 8,601 participants over the age of 30 were included in the study, of whom 3,954 were diagnosed with periodontitis. Table 1 presents participant characteristics categorized by periodontal status. Compared to those without periodontitis, individuals in the case group showed higher distributions of variables such as age, TP, TG, AST, BUN, SUA, and SCr, along with lower distributions of TC and HDL-C. Additionally, BMI, ALT, and calcium levels did not significantly differ between the periodontitis and control groups. Factors associated with a higher likelihood of periodontitis included being male, non-Hispanic White, having a higher education level, being married or living with a partner, belonging to the middle-income group, smoking, and alcohol consumption.

**Table 1:** Characteristics of 8,601 participants of this study.

Variables	Total	Non-periodontitis	Periodontitis	*p*-value
Number (%)	8,601	4,647 (54.03%)	3,954 (45.97%)	
Age, years	52.17 ± 14.26	48.31 ± 13.58	56.71 ± 13.68	<0.001
Gender (%)				<0.001
Male	4,331 (50.35%)	1,976 (42.52%)	2,355 (59.56%)	
Female	4,270 (49.65%)	2,671 (57.48%)	1,599 (40.44%)	
Race (%)				<0.001
Mexican American	1,179 (13.71%)	501 (10.78%)	678 (17.15%)	
Other Hispanic	813 (9.45%)	442 (9.51%)	371 (9.38%)	
Non-Hispanic White	3,968 (46.13%)	2,433 (52.36%)	1,535 (38.82%)	
Non-Hispanic Black	3,968 (46.13%)	736 (15.84%)	965 (24.41%)	
Other race	940 (10.93%)	535 (11.51%)	405 (10.24%)	
Education attainment (%)				<0.001
< high school	1,860 (21.63%)	650 (13.99%)	1,210 (30.60%)	
High school	1,870 (21.74%)	851 (18.31%)	1,019 (25.77%)	
> high school	4,871 (56.63%)	3,146 (67.70%)	1,725 (43.63%)	
Marital status (%)				<0.001
Married or living with a partner	5,589 (64.98%)	3,161 (68.02%)	2,428 (61.41%)	
Living alone	3,012 (35.02%)	1,486 (31.98%)	1,526 (38.59%)	
PIR (%)				<0.001
Low	1,613 (18.75%)	653 (14.05%)	960 (24.28%)	
Middle	1,613 (18.75%)	2,217 (47.71%)	2,211 (55.92%)	
High	2,560 (29.76%)	1,777 (38.24%)	783 (19.80%)	
Smoking status (%)				<0.001
Yes	3,847 (44.73%)	1,685 (36.26%)	2,162 (54.68%)	
No	4,754 (55.27%)	2,962 (63.74%)	1,792 (45.32%)	
Drinking status (%)				0.018
Yes	6,350 (73.83%)	3,479 (74.87%)	2,871 (72.61%)	
No	2,251 (26.17%)	1,168 (25.13%)	1,083 (27.39%)	
Hypertension (%)				<0.001
Yes	3,278 (38.11%)	1,499 (32.26%)	1,779 (44.99%)	
No	3,278 (38.11%)	3,148 (67.74%)	2,175 (55.01%)	
Diabetes				<0.001
Yes	1,074 (12.49%)	398 (8.56%)	676 (17.10%)	
No	7,287 (84.72%)	4,133 (88.94%)	3,154 (79.77%)	
Borderline	240 (2.79%)	116 (2.50%)	124 (3.14%)	
BMI (Kg/m2)	29.44 ± 6.75	29.36 ± 6.76	29.53 ± 6.73	0.095
TP (g/L)	71.20 ± 4.78	70.99 ± 4.54	71.45 ± 5.03	<0.001
TC (mmol/L)	5.08 ± 1.06	5.11 ± 1.04	5.05 ± 1.09	0.004
TG (mmol/L)	1.78 ± 1.33	1.72 ± 1.29	1.85 ± 1.38	<0.001
HDL-C (mmol/L)	1.37 ± 0.42	1.39 ± 0.41	1.34 ± 0.42	<0.001
ALT (U/L)	25.97 ± 18.97	25.84 ± 17.66	26.12 ± 20.41	0.736
AST (U/L)	26.26 ± 19.30	25.96 ± 21.39	26.61 ± 16.51	0.022
BUN (mmol/L)	4.77 ± 1.96	4.59 ± 1.75	4.99 ± 2.17	<0.001
SUA (mmol/L)	325.68 ± 84.24	317.16 ± 81.65	335.71 ± 86.13	<0.001
SCr (umol/L)	80.01 ± 33.24	77.14 ± 27.63	83.38 ± 38.54	<0.001
Calcium (mmol/L)	2.35 ± 0.09	2.35 ± 0.09	2.35 ± 0.09	0.711

PIR, family poverty-to-income ratio; BMI, body mass index; TP, total protein; TC, total cholesterol; TG, triglycerides; HDL-C, high-density lipoprotein cholesterol; ALT, alanine aminotransferase; AST, aspartate aminotransferase; BUN, blood urea nitrogen; SUA, serum uric acid; SCr, serum creatinine.

### 3.2 The relationship between serum calcium level and periodontitis

As shown in Table 2, the study results demonstrated an inverse association between serum calcium levels and periodontitis risk. When serum calcium levels were categorized into quartiles, both minimally and fully adjusted models indicated a negative correlation with periodontitis (*p* < 0.05). Specifically, individuals in the highest quartile of calcium had an 18% lower risk of periodontitis compared to those in the lowest quartile (OR = 0.82, 95% CI: 0.71–0.95, *p* = 0.0083; *p* for trend = 0.0057).

**Table 2:** OR (95% CI) and *p*-value for periodontitis risk across serum calcium level quartiles in NHANES 2009–2014.

Quartile of calcium					
	Quartile 1	Quartile 2	Quartile 3	Quartile 4	*p* for trend
Range (mmol/L)	<2.3	2.3–2.35	2.35–2.4	>2.4	
Model 1	1.0 [Reference]	0.93 (0.82, 1.05) 0.2442	0.88 (0.78, 1.00) 0.0541	0.95 (0.85, 1.07) 0.3958	0.9772
Model 2	1.0 [Reference]	0.92 (0.80, 1.06) 0.2495	0.83 (0.73, 0.95) 0.0089	0.85 (0.75, 0.97) 0.0151	0.0297
Model 3	1.0 [Reference]	0.95 (0.82, 1.10) 0.4783	0.84 (0.72, 0.97) 0.0195	0.82 (0.71, 0.95) 0.0083	0.0057

Model 1 included no covariate adjustments. Model 2 adjusted for age, gender, and race, while Model 3 included a more comprehensive adjustment for factors such as age, sex, race, educational level, marital status, PIR, smoking status, alcohol intake, hypertension, diabetes, BMI, TC, TG, HDL-C, ALT, AST, BUN, SUA, and SCr. OR, odds ratio; CI, confidence interval; PIR, family poverty-to-income ratio; BMI, body mass index; TP, total protein; TC, total cholesterol; TG, triglycerides; HDL-C, high-density lipoprotein cholesterol; ALT, alanine aminotransferase; AST, aspartate aminotransferase; BUN, blood urea nitrogen; SUA, serum uric acid; SCr, serum creatinine.

### 3.3 Subgroup analysis

**Figure 2:**
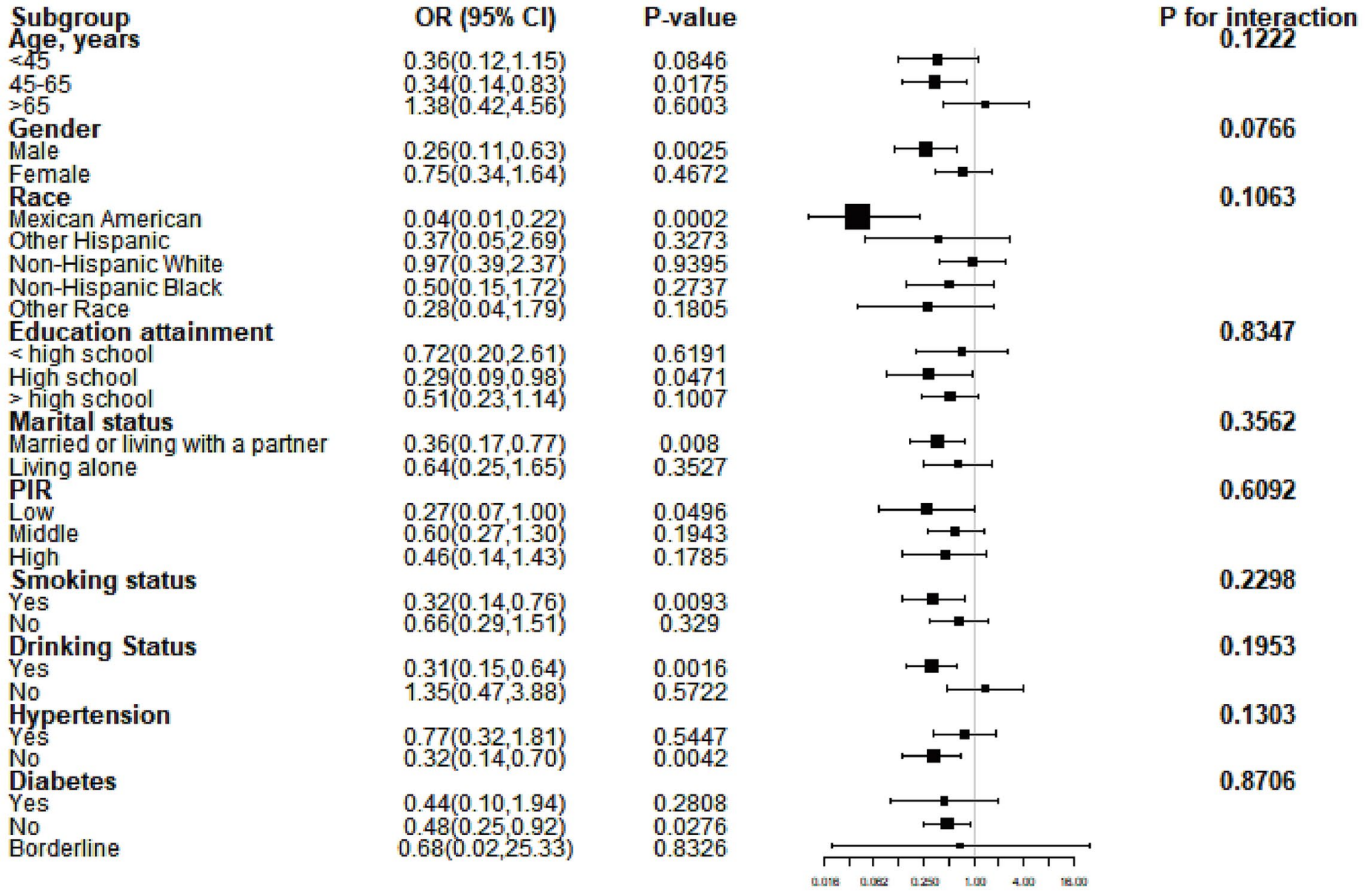
Subgroup analysis results based on model 3. OR, odds ratio; CI, confidence interval; PIR, poverty-to-income ratio.

Subgroup analysis was performed to evaluate the consistency of the relationship between serum calcium levels and periodontitis across different subgroups. Our results indicated that the association between calcium levels and periodontitis did not vary by subgroup. As shown in Figure 2, none of the factors—such as age, gender, race, education level, marital status, PIR, smoking, alcohol consumption, hypertension, or diabetes—affected the inverse relationship between calcium and periodontitis (all *p* for interaction >0.05).

### 3.4 Smoothed curve fitting and threshold effect analysis

**Figure 3:**
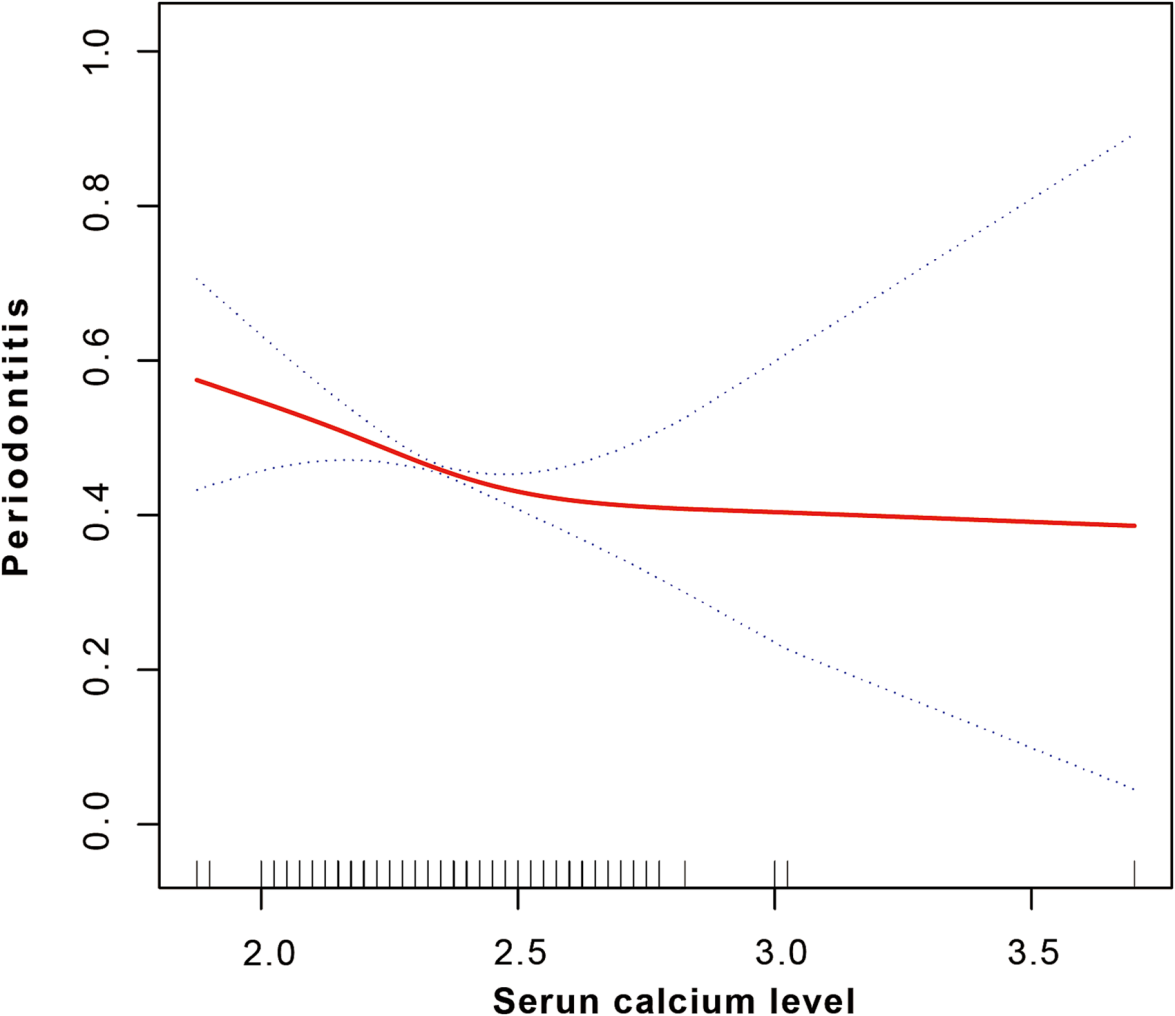
Smoothed fitting curve indicating a non-linear negative correlation between serum calcium level and periodontitis.

After adjusting for all covariates, a smoothed curve fitting was used to visually depict the relationship between serum calcium levels and periodontitis. This curve (Figure 3) showed a nonlinear inverse correlation between calcium levels and periodontitis. Threshold effect analysis further confirmed this nonlinear negative association (OR = 0.43, 95% CI = 0.24–0.78, *p* = 0.0057), although no significant inflection point was found at 2.48, with *p*-value of Logarithm likelihood ratio test = 0.094, as presented in Table 3.

**Table 3:** Threshold effect analysis of serum calcium level on periodontitis.

Periodontitis	Adjusted OR (95% CI)	*p*-value
Fitting by standard linear model	0.43 (0.24, 0.78)	0.0057
Fitting by two-piecewise linear model*		
Turning point (2.48 mmol/L)		
Calcium<2.48 mmol/L	0.31 (0.16, 0.63)	0.0012
Calcium>2.48 mmol/L	2.40 (0.31, 18.48)	0.3997
LRT test		0.094

*Adjusted by age, sex, race, educational level, marital status, PIR, smoking status, alcohol intake, hypertension, diabetes, BMI, TC, TG, HDL-C, ALT, AST, BUN, SUA, and SCr. OR, odds ratio; CI, confidence interval; LRT, Logarithm likelihood ratio.

## 4 Discussion

In this study, we investigated the association between serum calcium levels and periodontitis in a U.S. adult population, utilizing data from the NHANES 2009–2014. Our findings demonstrated a significant inverse association, with higher serum calcium levels linked to a reduced risk of periodontitis. This relationship persisted across multiple subgroups and adjustment models, and the nonlinear analysis further supported this inverse association without identifying a critical threshold point. These results suggest that maintaining adequate calcium levels could play a role in periodontitis prevention and inform both diagnostic and therapeutic strategies for periodontal health.

Previous epidemiological studies have explored the relationship between calcium levels and periodontal disease, with several studies supporting the potential protective role of calcium against periodontitis (18). For example, a study found that individuals taking vitamin D and calcium supplements showed trends of improved periodontal health, including shallower probing depths, less attachment loss, and reduced alveolar crest height loss, compared to non-supplement users (19). Research discovered that higher intakes of calcium, casein, and whey protein were associated with a lower likelihood of severe periodontitis, while vitamin D intake alone showed no significant association, highlighting the potential preventive role of calcium and protein-rich foods in periodontal health. Studies have shown that calcium supplementation may lower the risk of periodontal disease progression (28). Calcium and vitamin D supplementation had a modest positive effect on periodontal health over 1 year, though consistent dental care improved periodontal parameters in all participants, highlighting the need for further randomized trials (20). Research by Adegboye et al. and others has reported that individuals with higher calcium intake had a lower prevalence of periodontal disease among Danish adults, emphasizing calcium’s influence on maintaining periodontal health (28, 29). A systematic review explores the salivary ionomic profile in periodontitis compared to healthy controls. Findings indicate elevated levels of sodium and potassium in periodontitis patients, with calcium, copper, and manganese also showing increased trends in some studies (30). These findings contribute to a growing body of literature suggesting that calcium is a modifiable factor in periodontal disease risk.

The protective role of calcium against periodontitis can be attributed to several key biological mechanisms. First, calcium is essential for bone mineralization and structural integrity, directly influencing the alveolar bone that supports teeth (31, 32). Sufficient calcium levels are crucial in maintaining bone density, preventing resorption, and reducing susceptibility to bone loss, which is a primary characteristic of periodontitis (33, 34). Additionally, calcium plays a role in the mineralization of dental tissues, enhancing periodontal stability (33, 34). Beyond structural support, calcium also plays a critical role in cellular signaling and immune response regulation (35). It acts as a secondary messenger in various immune cells, influencing the activity of leukocytes and macrophages that are involved in inflammation and immune response (35). Adequate calcium levels help stabilize these cell membranes, potentially mitigating exaggerated inflammatory responses in the periodontal tissue, which could otherwise lead to tissue breakdown and disease progression (35). Calcium’s involvement in reducing inflammatory cytokines, such as IL-1β and TNF-*α*, may further decrease periodontal tissue inflammation, thereby limiting disease progression and supporting tissue healing (31, 36). A study focused on the efficacy of calcium hydroxide (Ca[OH]2) as an intracanal medication in reducing endotoxin levels in infected root canals, a condition linked to periodontal health. The findings reveal that Ca[OH]2 significantly decreased lipopolysaccharide levels, especially when combined with antimicrobial agents like chlorhexidine. The paper highlights its role in modulating inflammatory mediators, emphasizing its potential relevance to systemic effects linked to periodontitis (37). Finally, calcium may aid in vascular health, as calcium-dependent signaling is crucial for vascular tone and endothelial function, potentially affecting the blood supply and healing capacity of periodontal tissues (38).

Considering current evidence, calcium intake appears to have potential applications in periodontitis prevention, particularly in at-risk populations. Increasing dietary calcium intake or considering supplementation in individuals with low serum calcium levels could be beneficial in maintaining periodontal health. Given the positive outcomes observed in this study, further investigation is warranted to evaluate calcium’s role in periodontal health across diverse populations and to explore its potential as a preventive measure in clinical practice.

This study has several notable strengths. A primary strength is the use of data from the NHANES database, a large, nationally representative survey that ensures robust and generalizable findings across diverse demographic groups. Additionally, the study design included a thorough adjustment for potential confounders, such as lifestyle factors (smoking, alcohol use), demographic variables (age, gender, income), and health conditions (hypertension, diabetes), which strengthens the reliability of the observed associations. The use of advanced statistical techniques, including nonlinear analysis and threshold effect analysis, allowed for a nuanced understanding of the calcium-periodontitis relationship, providing insights that extend beyond a simple linear association.

However, there are also limitations to consider. The cross-sectional nature of the study limits the ability to draw causal inferences between serum calcium levels and periodontitis risk. Longitudinal studies would be required to determine whether calcium deficiency directly contributes to periodontitis development over time. Another limitation is the reliance on self-reported data for some variables, such as smoking and alcohol use, which may introduce recall bias. Additionally, unmeasured confounders, such as dietary habits and genetic predispositions, could influence both serum calcium levels and periodontitis risk, potentially impacting the observed association. Lastly, while this study highlights the potential importance of calcium in periodontal health, the exact optimal serum calcium levels and the role of dietary calcium intake versus serum calcium concentration remain to be fully clarified. Future research should aim to address these limitations through well-designed, longitudinal studies with precise measurement of dietary calcium intake and more detailed biochemical assessments.

## 5 Conclusion

This study found an inverse relationship between serum calcium levels and periodontitis risk, suggesting that adequate calcium may help protect against periodontal disease. Increasing calcium intake could benefit individuals at higher risk, particularly those with low serum calcium. While promising, further research is needed to confirm causality and determine optimal calcium levels for periodontal health, potentially informing future preventive and therapeutic strategies.

## Data Availability

The original contributions presented in the study are included in the article/Supplementary material, further inquiries can be directed to the corresponding author.

## Footnotes

^1^ https://cloud.r-project.org

^2^ http://www.empowerstats.net
