# Supplementary material for: Association of serum calcium level with periodontitis: a cross-sectional study from NHANES 2009–2014
Source: Front Nutr. 2025 Jan 7;11:1520639. doi: 10.3389/fnut.2024.1520639 (PMC11747520; doi:10.3389/fnut.2024.1520639)
Supplement: Supplementary file 1 [file Table_1.docx]

Supplementary Table 1: The definition of periodontitis based on CDC/AAP

| Category | Definition |
| --- | --- |
| Healthy | No evidence of mild, moderate, or severe periodontitis |
| Mild periodontitis | ≥2 interproximal sites with AL ≥3 mm, and ≥2 interproximal sites with PD ≥4 mm (not on same tooth) or 1 site with PPD ≥5 mm |
| Moderate periodontitis | ≥2 interproximal sites with PPD ≥ 5 mm, not on the same tooth, or ≥2 interproximal sites with AL ≥ 4 mm, not on the same tooth |
| Severe periodontitis | ≥2 interproximal sites with AL ≥ 6 mm, not on the same tooth, and ≥1 interproximal site with PPD ≥ 5 mm |
